# Structure Elucidation and Cholinesterase Inhibition Activity of Two New Minor Amaryllidaceae Alkaloids

**DOI:** 10.3390/molecules26051279

**Published:** 2021-02-26

**Authors:** Jana Maříková, Abdullah Al Mamun, Latifah Al Shammari, Jan Korábečný, Tomáš Kučera, Daniela Hulcová, Jiří Kuneš, Milan Malaník, Michaela Vašková, Eliška Kohelová, Lucie Nováková, Lucie Cahlíková, Milan Pour

**Affiliations:** 1 Department of Bioorganic and Organic Chemistry, Faculty of Pharmacy, Charles University, Heyrovskeho 1203, 500 05 Hradec Kralove, Czech Republic; marikoj2@faf.cuni.cz (J.M.); kunes@faf.cuni.cz (J.K.); 2 ADINACO Research Group, Department of Pharmaceutical Botany, Faculty of Pharmacy, Charles University, Heyrovskeho 1203, 500 05 Hradec Kralove, Czech Republic; almamuna@faf.cuni.cz (A.A.M.); alshamml@faf.cuni.cz (L.A.S.); hulcovd@faf.cuni.cz (D.H.); kohelove@faf.cuni.cz (E.K.); cahlikova@faf.cuni.cz (L.C.); 3 Department of Toxicology and Military Pharmacy, University of Defence, Trenesska 1575, 500 05 Hradec Kralove, Czech Republic; jan.korabecny@fnhk.cz (J.K.); tomas.kucera2@unob.cz (T.K.); 4 Biomedical Research Centre, University Hospital Hradec Králové, Sokolska 581, 500 05 Hradec Kralove, Czech Republic; 5 Department of Pharmacognosy, Faculty of Pharmacy, Charles University, Heyrovskeho 1203, 500 05 Hradec Kralove, Czech Republic; 6 Department of Natural Drugs, Faculty of Pharmacy, Masaryk University, Palackeho trida 1946/1, 612 00 Brno, Czech Republic; milan.malanik@seznam.cz; 7 Department of Chemistry, Faculty of Science, University of Hradec Kralove, Rokitanskeho 62, 500 03 Hradec Kralove, Czech Republic; michaela.vaskova@uhk.cz; 8 Department of Analytical Chemistry, Faculty of Pharmacy, Charles University, Heyrovskeho 1203, 500 05 Hradec Kralove, Czech Republic; novakoval@faf.cuni.cz

**Keywords:** Amaryllidaceae, 9-*O*-demethyllycorenine, narciabduliine, Alzheimer’s disease

## Abstract

Two new minor Amaryllidaceae alkaloids were isolated from *Hippeastrum* × *hybridum* cv. Ferrari and *Narcissus pseudonarcissus* cv. Carlton. The chemical structures were identified by various spectroscopic (one- and two-dimensional (1D and 2D) NMR, circular dichroism (CD), high-resolution mass spectrometry (HRMS) and by comparison with literature data of similar compounds. Both isolated alkaloids were screened for their human acetylcholinesterase (*h*AChE) and butyrylcholinesterase (*h*BuChE) inhibition activity. One of the new compounds, a heterodimer alkaloid of narcikachnine-type, named narciabduliine (**2**), showed balanced inhibition potency for both studied enzymes, with IC_50_ values of 3.29 ± 0.73 µM for *h*AChE and 3.44 ± 0.02 µM for *h*BuChE. The accommodation of **2** into the active sites of respective enzymes was predicted using molecular modeling simulation.

## 1. Introduction

Amaryllidaceae alkaloids (AAs), exclusively produced by Amaryllidaceae plants, are structurally unique sources of biologically active compounds. Analgesic, anti-inflammatory, antimalarial, antitumor, antimicrobial, and cholinesterase-inhibitory activities have been reported for these isoquinoline derivatives [1,2,3,4,5]. The most known AA is galantamine, which has been approved by the Food and Drug Administration (FDA) for the treatment of mild to moderate stages of Alzheimer’s disease, under the name Razadyne© [6].

Our research group recently isolated several new AAs with impressive acetylcholinesterase (AChE, EC: 3.1.1.7) and/or butyrylcholinesterase (BuChE, EC: 3.1.1.8) inhibitory potential from various Amaryllidaceae plants [7,8]. Unfortunately, some of these alkaloids are present in plants in low concentrations, but they can serve as a structural scaffold for inspiration in the design and development of drug candidates. For this reason, we continued the isolation of minor AAs from mother liquors of already studied plant species.

## 2. Results and Discussion

**Figure 1:**
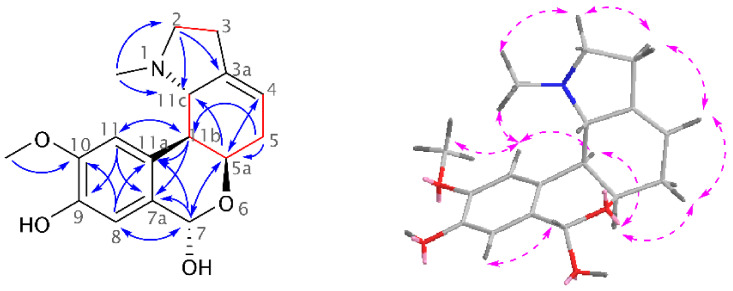
Structural elucidation of **1** showing key gCOSY, gH2BC (red lines), gHMBCAD (blue arrows), and NOESY (pink dashed arrows) correlations.

A new homolycorine-type AA, 9-*O*-demethyllycorenine (**1**), was isolated as a white amorphous solid from the mother liquor of *Hippeastrum* × *hybridum* cv. Ferrari, using preparative TLC. Since this alkaloid is a 9-*O*-demethyl analogue of the already described lycorenine, the name of this new substance was derived from it. The ESI-HRMS of **1** showed a molecular ion peak [M + H]^+^ at *m*/*z* 304.1550, corresponding to the formula C_17_H_21_NO_4_^+^ (calculated 304.1543, Figure S1). The lycorenine-type skeleton was proven in the ^1^H-NMR spectrum by the presence of two singlets of a 1,2,4,5-tetrasubstituted benzene ring (*δ*_H_ 6.94, H-8; 6.91, H-11), one deshielded singlet of a benzylic proton (*δ*_H_ 6.01, H-7), a multiplet of olefinic protons (*δ*_H_ 5.50–5.46, H-4), and so on. Compared to the resonances of three lycorenine methyls, only two signals of the methyl groups were recognized in the ^1^H-NMR spectrum of **1**: the deshielded *O*-methyl (*δ*_H_ 3.89, s) and the methyl of the *N*CH_3_ group (*δ*_H_ 2.10, s) (see Figure S3, Supplementary Materials). Employing two-dimensional (2D) NMR experiments, the constitution of **1** was confirmed (Figure 1). The crucial position of the *O*CH_3_ was determined by interactions in the adiabatic gHMBC experiment (gHMBCAD) and supported by a cross-peak of its methyl protons and H-11 in the NOESY spectrum. This experiment was used for the establishment of the relative configuration as follows: the cross-peak of H-5a/H-11b in the NOESY spectrum presented in the *cis* position for these protons, and no through-space interaction of H-5a/H-7 and H-11b/H-11c determined H-11b/H-11c with a *trans* orientation. Moreover, H-11b coupled to H11c with ^3^*J* = 9.5 Hz, which corresponds to a *trans*-pseudo-diaxial position. Then, the absolute configuration was determined as (5a*R*,7*S*,11b*S*,11c*S*) using chiroptical methods and comparison with published data of similar compounds. The electronic circular dichroism (ECD) spectrum of **1,** with negative Cotton effects at 237 nm and 282 nm, was in good agreement with that of lycorenine (see Figure S2, Supplementary Materials). The dextrorotatory value is in accordance with those found for other alkaloids possessing a lycorenine skeleton, such as lycorenine, nerinine, and oduline [9].

**Figure 2:**
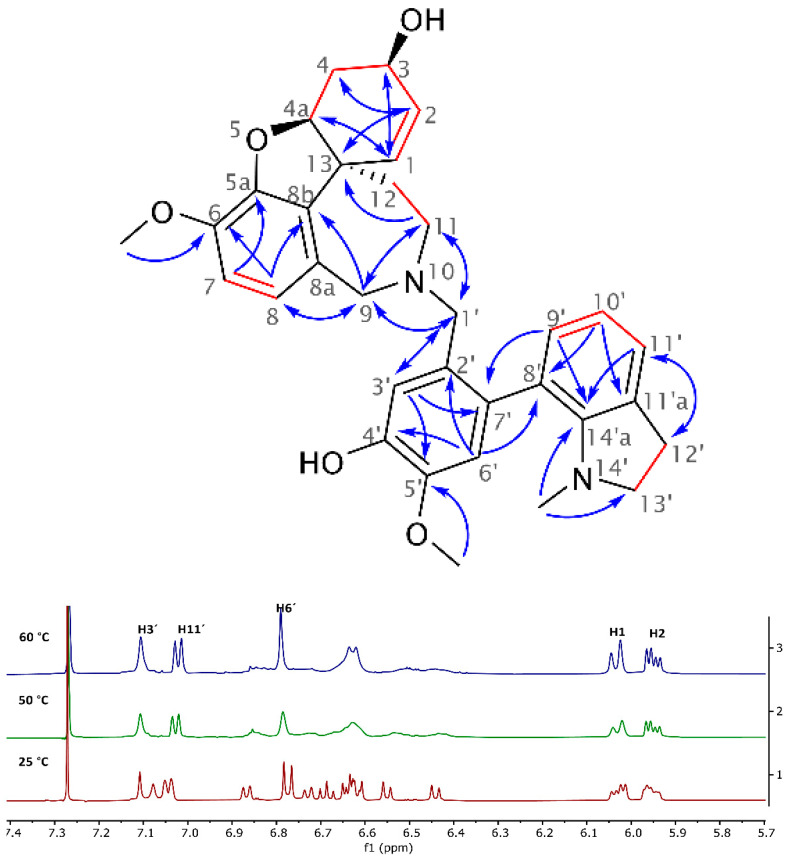
Key NMR correlations of **2** (red lines show gCOSY and gH2BC correlations, and blue arrows indicate interactions in the gHMBCAD experiment) and the aromatic region of its ^1^H-NMR spectrum at different temperatures, which demonstrates coalescence of highlighted signals during dynamic NMR analysis (measured in CDCl_3_).

The novel heterodimeric alkaloid **2** isolated from *Narcissus pseudonarcissus* cv. Carlton was obtained as a white amorphous solid and named narciabduliine. The ESI-HRMS of **2** showed a molecular ion peak [M + H]^+^ at *m*/*z* 541.2707, corresponding to the formula C_33_H_37_N_2_O_5_^+^ (calculated 541.2697). According to our previous structural elucidations of narcikachnine-type AAs, narciabduliine **2** showed great structural similarity with narcieliine [8,10,11]. A diastereomeric mixture of atropisomers in a 1:1.1 ratio was identified as well (see Figure S11, Supplementary Materials). The individual signals of protons and carbons were unambiguously assigned into specific groups employing gHSQC experiment. Unfortunately, due to some overlapping signals of the diastereomers, it was not possible to separate the corresponding signals for each isomer. Comparable with narcieliine, galanthindole, and galantamine, fragments with an N10-C2′ methylene bridge adjustment were determined in **2** using gHMBCAD, gCOSY, and H2BC experiments (Figure 2). The only structural difference from narcieliine was found in a C-3′,C-4′-substitution in the galanthindole moiety. A protonated *sp*^2^-carbon was identified as C-3′ (*δ*_C_ 114.8 and 114.6; *δ*_H_ 7.11, s and 7.08, s), and the C-4′-substituent was determined as a hydroxyl group (*δ*_C_ 145.0 and 144.8). The biological effect of this alteration is discussed below.

Dynamic NMR analysis was performed to prove the conformational phenomenon of these two stereoisomers. However, the energy levels of the coalescence were very similar to those of the decomposition in CDCl_3_ solution at 60 °C. The stability of **2** is much lower than that of narcieliine. Despite this, indisputable proof of atropisomerism was demonstrated (see Figure 2). All variable-temperature data are shown in the Supplementary Materials (Figure S19).

As a part of our studies on searching for new compounds from natural sources for the potential treatment of Alzheimer’s disease, both new alkaloids were screened for their *h*AChE/*h*BuChE inhibition potency. The *h*AChE/*h*BuChE inhibitory activities of **1** and **2** were initially screened at a concentration of 100 µM. Compound **2** displayed inhibition ability >90% against both types of cholinesterase at the screening concentration; thus, the IC_50_ values were determined (Table 1). In contrast, **1** was almost devoid of cholinesterase inhibitory activity.

**Figure 3:**
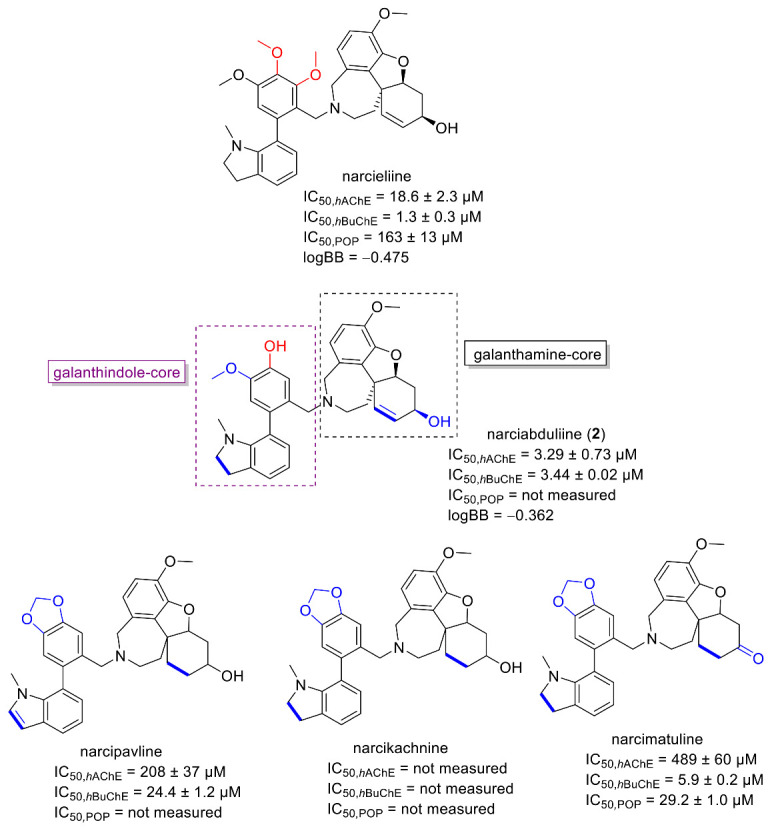
Structures of **2** and of recently isolated narcikachnine-type Amaryllidaceae alkaloids (structural differences of discussed alkaloids and **2** are marked in blue and red).

**Figure 4:**
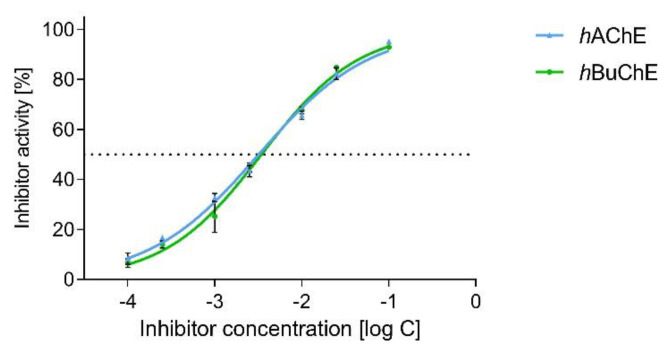
Dose-dependent plot for *h*AChE and *h*BuChE of **2**.

Interestingly, dual *h*AChE/*h*BuChE inhibition potency was shown by the alkaloid narciabduliine (**2**). This structural type of AA is a combination of galantamine and galanthindole cores. Recently, several alkaloids of this structural type isolated from Amaryllidaceae plants by our group demonstrated interesting *h*BuChE inhibition activity [7,8,10,11]. Compared with previously isolated narcikachnine-type AAs, the new alkaloid, narciabduliine (**2**), demonstrated balanced *h*AChE/*h*BuChE inhibition activity with an IC_50_ value of 3.24 ± 0.73 µM for *h*AChE and 3.44 ± 0.02 µM for *h*BuChE (Figure 3 and Figure 4, respectively). Narcipavline and narcimatuline displayed only weak *h*AChE inhibition potency (IC_50_ > 200 µM for both compounds), and narcikachnine was not studied due to its isolation in low quantity (Figure 3). The closest match in the structure of **2** and its biological activity was observed for narcieliine, recently isolated from *Zephyranthes citrina* [8]. As discussed in the structural elucidation of **2**, the only difference is the substitution of a benzene ring moiety; narcieliine has three methoxy groups of the 1,2,3,4,5-pentasubstituted benzene ring all present**,** whereas the new alkaloid **2** contains only one methoxy and one hydroxy group in a 1,2,4,5-tetrasubstituted ring (Figure 3). This small change in the structure is related to an interesting increase in *h*AChE inhibition potency of **2** and only a small decline in *h*BuChE activity compared with narcieliine (Figure 3). Other discussed alkaloids of this group contain a benzo[*d*][1,3]dioxol moiety instead (see Figure 3). 

To elucidate the binding mode of **2** in the active sites of *h*AChE/*h*BuChE, a molecular modeling study was applied. The template of *h*AChE complexed with galantamine from protein data bank (PDB ID: 4EY6) [12] was used due to the structural resemblance of **2** to galantamine. For docking studies with *h*BuChE, we applied the crystal structure with another reversible inhibitor, namely, tacrine [13]. The critical aspect in both cases was placed onto the high-resolution region. Indeed, *h*AChE and *h*BuChE enzymes embedded with the respective ligands were solved at 2.4 Å and 2.1 Å, respectively.

**Figure 5:**
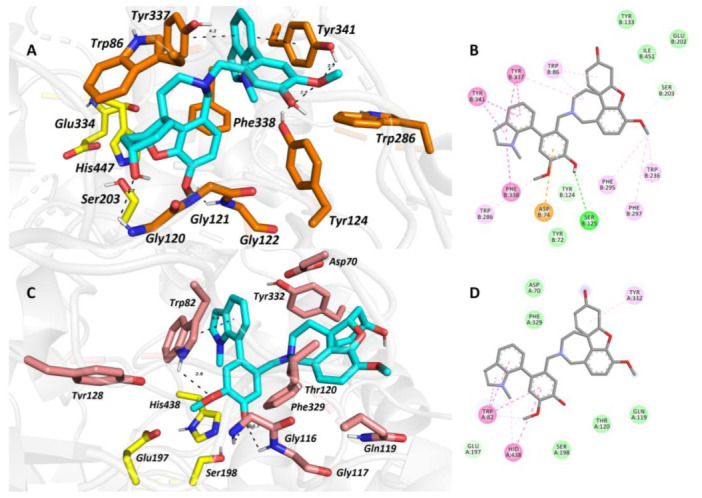
The top-scored docking poses of **2** in the *h*AChE (**A**,**B**; PDB ID: 4EY6) and *h*BuChE (**C**,**D**; PDB ID: 4BDS) active sites. Spatial orientation for each ligand is presented as three-dimensional (**A**,**C**) and two-dimensional (**B**,**D**) diagrams, respectively. The ligand is displayed in light blue (**A**,**C**); important amino-acid residues responsible for ligand anchoring are shown in orange for *h*AChE and salmon for *h*BuChE. Catalytic triad residues are displayed in yellow (**A**,**C**). Important interactions are rendered with black dashed lines; distances are measured in angstroms (Å). The rest of the receptor is displayed in light-gray cartoon conformation (**A**,**C**). Panels (**A**) and (**C**) were created with the PyMOL Molecular Graphics System, Version 2.4.1, Schrödinger, LLC. Two-dimensional (2D) diagrams (**B**,**D**) were created with Dassault Systèmes 2016, BIOVIA, Discovery Studio Visualizer, v 17.2.0.16349, San Diego, CA, USA.

The top-scored docking pose of **2** in the cavity of *h*AChE (Figure 5A,B) revealed that galantamine moiety is bound to the catalytic anionic site (CAS), whereas the galanthindole core is lodged peripherally. In line with the crystal structure of galantamine in *h*AChE [12], the cyclohex-2-en-1-ol moiety is located in the vicinity of Trp86. Interaction of **2** with the oxyanion hole, formed by Gly120–122 residues, is mediated via hydrogen bonds to both the hydroxyl group of cyclohex-2-en-1-ol moiety and the methoxy group of the phenyl ring. In the crystal structure of galantamine, this complexation is generated by water-mediated hydrogen bonds to methoxybenzene only. Furthermore, the hydroxyl group of **2** seems to stand aside from the Ser203 residue. We deem that the rotation of Ser203 to a plausible interaction with hydroxyl moiety may occur, enabling hydrogen salt formation. The presence of the galanthindole moiety in **2** made it so that the 1-methyl-2,3-dihydro-1*H*-indole moiety is sandwiched between Tyr337 (4.2 Å) and Tyr341 (3.9 Å) by parallel π–π stackings, hampering the hydrogen-bond donor contact between the hydroxyl group of Tyr337 to nitrogen from the azepine ring of **2**. In general, the overall topology of **2** in the *h*AChE shares a high similarity to that of galantamine, but the galanthindole moiety allowed spanning **2** into the peripheral anionic site (PAS) of the enzyme.

The best scoring pose of **2** in the active site of *h*BuChE (Figure 5C,D) presumes that the ligand adopted inverse accommodation to that observed for the **2**–*h*AChE complex. Indeed, the galanthindole moiety of **2** is buried inside the enzyme’s cavity, whereas the galantamine core of **2** is situated distally. Such a binding pose is in accordance with the more sterically demanding properties of the galanthindole moiety and the bowl-shaped *h*BuChE enzyme that can accommodate bulkier ligands than the narrow gorge of *h*AChE [14]. The critical interactions for the galanthindole moiety within the CAS region of *h*BuChE can be defined as follows: (i) distorted π–π stacking between 1-methyl-2,3-dihydro-1*H*-indole moiety of **2** and Trp82 (4.6 Å), (ii) hydrogen bond between oxygen from methoxy group and hydrogen at 1-methyl-2,3-dihydro-1*H*-indole moiety, (iii) two hydrogen bonds between phenolic hydroxyl functionality of **2** and glycine residues 116 and 117 (2.7 Å and 2.7 Å), and (iv) plausible hydrogen contact to Ser198 (3.1 Å) from the catalytic triad. The galantamine moiety seems to contribute less to the overall ligand anchoring, forming hydrophobic contacts with Tyr332 and Gln119 only.

The prediction of CNS availability is critical for drugs developed for neurodegenerative diseases; thus, we calculated the logBB value, which predicts the logarithmic ratio between the concentration of a compound in the brain (C*_brain_*) and blood (C*_blood_*). Compounds with a logBB >0.3 can readily penetrate the blood–brain barrier (BBB), whereas those with logBB <−1.0 are only poorly distributed to the brain [15]. The obtained logBB value of −0.362 for **2** indicates that the new alkaloid should be able to reach the target area in the CNS. Due to the isolation of a limited amount, **2** was not studied for its in vitro permeability (Pe) through biological membranes. However, if these obtained logBB values of **2** and narcieliine are compared (Figure 3), we can assume that **2** could cross the blood–brain barrier (BBB) via passive diffusion, since the in vitro permeability of narcieliine (*Pe* = 14.1 ± 1.0 × 10^−6^ cm·s^−1^) indicated centrally active crossing of the BBB via passive diffusion [8]. Summarizing the obtained and reported results, it can be concluded that narcikachnine-type AAs represent an interesting structural scaffold with cholinesterase inhibition potential.

## 3. Materials and Methods

### 3.1. General Experimental Procedures

All solvents were treated using standard techniques before use. All reagents and catalysts were purchased from Sigma Aldrich, Prague, Czech Republic, and used without purification. NMR spectra were recorded in CDCl_3_ on a VNMR S500 (Varian, Palo Alto, CA, USA) spectrometer operating at 500 MHz for ^1^H and 125.7 MHz for ^13^C at ambient temperature. The residual signal of CHCl_3_ (*δ* 7.26 ppm) was a reference for ^1^H-NMR spectra, and the central signal of the CDCl_3_ signals (*δ* 77.0 ppm) was used as a reference for proton-decoupled ^13^C-NMR spectra. The coupling constants (*J*) are given in Hz, and the chemical shifts are reported in ppm. For unambiguous assignment of ^1^H- and ^13^C-NMR signals, 2D NMR experiments, namely, gCOSY, gHSQC, gHMBCAD, gH2BC, and NOESY, were measured using standard parameter settings and standard pulse programs provided by the manufacturer of the spectrometer. Dynamic NMR analysis was performed at 25 °C, 50 °C, and then 60 °C. ESI-HRMS data were obtained with a Waters Synapt G2-Si hybrid mass analyzer of a quadrupole time-of-flight (Q-TOF) type, coupled to a Waters Acquity I-Class UHPLC system. The EI-MS were obtained on an Agilent 7890A GC 5975 inert MSD operating in EI mode at 70 eV (Agilent Technologies, Santa Clara, CA, USA). A DB-5 column (30 m × 0.25 mm × 0.25 μm, Agilent Technologies, Santa Clara, CA, USA) was used with the following temperature program: 100–180 °C at 15 °C/min, 1 min hold at 180 °C, 180–300 °C at 5 °C /min, and 5 min hold at 300 °C; the detection range was *m*/*z* 40–600. The injector temperature was 280 °C. The flow rate of carrier gas (helium) was 0.8 mL/min. A split ratio of 1:15 was used. UV and ECD spectra were recorded on a JASCO J-815 CD spectrometer. Compounds on the plate were observed under UV light (254 and 366 nm) and visualized by spraying with Dragendorff’s reagent. 

### 3.2. Isolation of Compound 1

The main procedure for the isolation of **1** from *Hippeastrum* × *hybridum* cv. Ferrari was described in [16]. Compound **1** (10 mg; 6/100) was isolated from the mother liquor (150 mg) of fraction **VI** using preparative TLC (To:EtOAc:Et_2_NH, 60:30:10).

9-*O*-Demethyllycorenine (**1**): white amorphous solid; [α]^24^_D_ = +60° (*c* = 0.10; MeOH); **ECD** (*c* = 0.10, MeOH) *λ*_max_ (Δε) 237 (−4.68), 282 (−1.49) nm; **^1^H-NMR** (500 MHz, CDCl_3_) δ: 6.94 (1H, s, H-8), 6.91 (1H, s, H-11), 6.01 (1H, s, H-7), 5.50–5.46 (1H, m, H-4), 4.37 (1H, dd, *J* = 5.5 Hz, *J* = 1.8 Hz, H-5a), 3.89 (3H, s, 10-OCH_3_), 3.15 (1H, ddd, *J* = 9.4 Hz, *J* = 6.7 Hz, *J* = 3.7 Hz, H-2), 2.73 (1H, d, *J* = 9.5 Hz, H-11c), 2.68–2.59 (1H, m, H-5), 2.53–2.46 (2H, m, H-3), 2.44 (1H, dd, *J* = 9.5 Hz, *J* = 1.8 Hz, H-11b), 2.39–2.29 (1H, m, H-5), 2.24 (1H, dt, *J* = 9.4 Hz, *J* = 9.4 Hz, H-2), 2.10 (3H, s, N1-CH_3_); **^13^C-NMR** (126 MHz, CDCl_3_) δ: 146.2 (C-10), 145.0 (C-9), 140.8 (C-3a), 129.9 (C-11a), 127.5 (C-7a), 115.7 (C-4), 113.2 (C-8), 112.0 (C-11), 91.8 (C-7), 67.3 (C-11c), 66.9 (C-5a), 56.9 (C-2), 56.2 (10-OCH_3_), 44.4 (C-11b), 44.3 (N1-CH_3_), 31.8 (C-5), 28.1 (C-3); **HRMS** *m*/*z* 304.1550 [M + H]^+^ (calculated for C_17_H_21_NO_4_^+^, 304.1543). See Supplementary Materials for NMR, ECD, and HRMS spectra.

### 3.3. Isolation of Compound 2

The main procedure used for the isolation of **2** from *Narcissus pseudonarcissus* cv. Carlton was described in [7]. Repetitive preparative TLC (CH_3_CN: EtOAc: NH_3_; 40:10:0.2) of subfraction **VIIIb** (355 mg) led to three subfractions VIIIb/1, VIIIb/2, and VIIIb/3. Compound **2** (11 mg) was obtained from subfraction VIIIb/2 after recrystallization from EtOAc. 

Narciabduliine (**2**): white amorphous solid; [α]^25^_D_ = −144 (*c* = 0.10; MeOH; *dr* 1:1.1); **^1^H-NMR** (500 MHz, CDCl_3_) *δ*: 7.11 (1H, s, H-3′), 7.08 (1H, s, H-3′), 7.04 (2H, d, *J* = 7.5 Hz, H-11′), 6.87 (1H, d, *J* = 7.5 Hz, H-9′), 6.78 (1H, s, H-6′), 6.77 (1H, s, H-6′), 6.73 (1H, d, *J* = 7.5 Hz, H-9′), 6.69 (1H, t, *J* = 7.5 Hz, H-10′), 6.66–6.59 (2H, m, H-7), 6.63 (1H, t, overlap, *J* = 7.5 Hz, H-10′), 6.55 (1H, d, *J* = 8.2 Hz, H-8), 6.44 (1H, d, *J* = 8.2 Hz, H-8), 6.03 (1H, d, overlap, *J* = 10.1 Hz, H-1), 6.02 (1H, d, overlap, *J* = 10.1 Hz, H-1), 5.98–5.93 (2H, m, H-2), 5.62 (2H, bs, OH), 4.54 (1H, bs, H-4a), 4.43 (1H, bs, H-4a), 4.14–4.07 (2H, m, H-3), 3.98 (1H, d, *J* = 14.8 Hz, H-9), 3.86 (1H, d, *J* = 15.1 Hz, H-9), 3.87 (3H, s, 5′-OCH_3_), 3.86 (3H, s, 5′-OCH_3_), 3.84 (6H, s, 6-OCH_3_), 3.71 (1H, d, *J* = 14.8 Hz, H-9), 3.56 (1H, d, *J* = 15.1 Hz, H-9), 3.49 (1H, d, *J* = 13.7 Hz, H-1′), 3.43 (2H, s, overlap, H-1′), 3.41 (1H, d, overlap, *J* = 13.7 Hz, H-1′), 3.30–3.17 (3H, m, H-13′), 3.17–3.05 (3H, m, H-13′, H-11), 3.02–2.89 (4H, m, H-12′, H-11), 2.87–2.77 (2H, m, H-12′, H-11), 2.69–2.61 (2H, m, H-4), 2.38 (2H, bs, OH), 2.22 (3H, s, N14′-CH_3_), 2.18 (3H, s, N14′-CH_3_), 2.01–1.90 (3H, m, H-4, H-12), 1.74 (1H, td, *J* = 13.6 Hz, *J* = 2.3 Hz, H-12), 1.49 (1H, d, *J* = 13.4 Hz, H-12), 1.35 (1H, d, *J* = 13.6 Hz, H-12); **^13^C-NMR** (125.7 MHz, CDCl_3_) *δ*: 150.4 (C-14′a), 150.3 (C-14′a), 145.8 (C-5a), 145.7 (C-5a), 145.0 (C-4′), 144.8 (C-4′), 144.6 (2× C-5′), 143.9 (2× C-6), 133.3 (2× C-8b), 132.0 (C-7′), 131.9 (C-7′), 131.0 (2× C-2′, 2× C-11′a), 130.5 (C-9′), 130.3 (2× C-8a, C-9′), 127.3 (2× C-1), 127.3 (2× C-2), 123.2 (2× C-11′), 123.0 (C-8′), 122.9 (C-8′), 122.0 (C-8), 121.9 (C-8), 117.8 (2× C-10′), 114.8 (C-3′), 114.6 (C-3′), 113.0 (C-6′), 112.9 (C-6′), 111.1 (C-7), 111.1 (C-7), 88.7 (C-4a), 88.6 (C-4a), 62.1 (C-3), 62.1 (C-3), 58.4 (C-9), 58.2 (C-9), 57.1 (C-13′), 57.0 (C-13′), 56.0 (2× 5′-OCH_3_), 56.0 (2× 6-OCH_3_), 54.6 (C-1′), 53.8 (C-1′), 51.9 (C-11), 51.1 (C-11), 48.3 (C-13), 48.2 (C-13), 38.6 (14′-CH_3_), 38.6 (14′-CH_3_), 34.7 (C-12), 34.0 (C-12), 29.9 (C-4), 29.9 (C-4), 28.6 (C-12′), 28.6 (C-12′); **HRMS** *m*/*z* 541.2707 [M + H]^+^ (calculated for C_33_H_37_N_2_O_5_^+^, 541.2697). See Supplementary Materials for NMR spectra and HRMS data.

### 3.4. hAChE and hBuChE Inhibition Assay

The *h*AChE and *h*BuChE activities of the studied compounds were determined using a modified method of Ellman with acetylthiocholine iodide (ATChI) and butyrylthiocholine iodide (BuTChI) as substrates, respectively, described recently by our group [17,18]. Briefly, 8.3 μL of either blood cell lysate or plasma dilutions (at least six different concentrations), 283 μL of 5 mM 5,5′-dithiobis-2-nitrobenzoic acid (DTNB), and 8.3 μL of the sample dilution in dimethyl sulfoxide (DMSO) (40 mM, 10 mM, 4 mM, 1 mM, 0.4 mM, and 0 mM) were added to the semi-microcuvette. The reaction was initiated by the addition of 33.3 μL 10 mM substrate (ATChI or BuTChI). The final proportion of DTNB and substrate was 1:1. The increase in absorbance (ΔA) at 436 nm for AChE and 412 nm for BuChE was measured for 1 min at 37 °C using a spectrophotometer (SynergyTM HT Multi-Detection Microplate Reader, Winooski, VT, USA). Each measurement was repeated six times for every concentration of enzyme preparation. The % inhibition was calculated according to the following formula:

(1) $$\%\;I=100-\left(100\times \frac{\Delta A_{Bl}}{\Delta A_{Sa}}\right)$$

where Δ*A_Bl_* is the increase in absorbance of the blank sample and Δ*A_Sa_* is the increase in absorbance of the measured sample. The inhibition potency of the tested compounds was expressed as an IC_50_ value (concentration of inhibitor which causes 50% cholinesterase inhibition).

### 3.5. Molecular Modeling Studies

Two structures of *h*AChE and *h*BuChE were gained from RCSB Protein Data Bank: PDB ID 4EY6 (crystal structure of *h*AChE) and 4BDS (crystal structure of hBuChE) [13]. All receptor structures were prepared using the DockPrep function of UCSF Chimera (version 1.4) and converted to pdbqt-files by AutodockTools (v. 1.5.6) [19,20]. Flexible residue selection was based on previous experience with *h*AChE, *h*BuChE, or the spherical region around the binding cavity [21,22]. Three-dimensional structures of ligands were built by Open Babel (v. 2.3.1), minimized by Avogadro (v 1.1.0), and converted to pdbqt-file format by AutodockTools [23]. The docking calculations were made by Autodock Vina (v. 1.1.2) with an exhaustiveness of 8 [24]. The calculation was repeated 20 times for each ligand and receptor and the best-scored result was selected for manual inspection. The visualization of enzyme–ligand interactions was prepared using the PyMOL Molecular Graphics System, Version 2.4.1 Schrödinger, LLC, Mannheim, Germany. The 2D diagrams were created with Dassault Systèmes 2016, BIOVIA, Discovery Studio Visualizer, v 17.2.0.16349, San Diego, CA, USA.

## Figures and Tables

**Table 1:** In vitro results of human acetylcholinesterase (*h*AChE) and butyrylcholinesterase (*h*BuChE) assays and calculation of logBB of reported alkaloids.

Compound	% Inhibition *h*AChE ± SEM ^a^	IC_50,_ *h*AChE ± SEM (µM) ^b^	% Inhibition *h*BuChE ± SEM ^a^	IC_50,_ *h*BuChE ± SEM (µM) ^b^	logBB ^c^
9-*O*-demethyllycorenine (**1**)	3.1 ± 1.2	>100	30.2 ± 0.8	>100	n.c.
narciabduliine (**2**)	94.7 ± 0.7	3.29 ± 0.73	94.1 ± 0.2	3.44 ± 0.02	−0.36
galantamine ^d^	98.8 ± 1.1	2.01 ± 0.14	68.2 ± 1.2	29.31 ± 3.49	0.05
eserine ^d^	99.8 ± 0.6	0.20 ± 0.0.01	99.9 ± 0.5	0.30 ± 0.01	−0.18

^a^ Tested at 100 µM compound concentration; ^b^ compound concentration required to decrease enzyme activity by 50%; the values are the mean ± SEM of three independent measurements, each performed in triplicate; ^c^ calculated at http://www.way2drug.com/geb/ (accessed on 10 October 2020); ^d^ reference compound; n.c. stands for not calculated.

## Data Availability

The data presented in this study are available within the article or supplementary materials.

## Supplementary Materials

Figure S1. ESI-HRMS spectrum of 9-*O*-demethyllycorenine (**1**); Figure S2. ECD data of 9-*O*-demethyllycorenine (**1**), and lycorenine; Figure S3. ^1^H-NMR spectrum of 9-*O*-demethyllycorenine (**1**) in CDCl_3_; Figure S4. ^13^C-NMR spectrum of 9-*O*-demethyllycorenine (**1**) in CDCl_3_; Figure S5. COSY spectrum of 9-*O*-demethyllycorenine (**1**); Figure S6. HSQC spectrum of 9-*O*-demethyllycorenine (**1**); Figure S7. HMBC spectrum of 9-*O*-demethyllycorenine (**1**); Figure S8. H2BC spectrum of 9-*O*-demethyllycorenine (**1**); Figure S9. NOESY spectrum of 9-*O*-demethyllycorenine (**1**); Figure S10. ESI-HRMS spectrum of narciabduliine (**2**); Figure S11. ^1^H-NMR spectrum of narciabduliine (**2**) in CDCl_3__;_ Figure S12. ^13^C-NMR spectrum of narciabduliine (**2**) in CDCl_3_; Figure S13. COSY spectrum of narciabduliine (**2**); Figure S14. HSQC spectrum (aromatic region) of narciabduliine (**2**); Figure S15. HSQC spectrum (aliphatic region) of narciabduliine (**2**); Figure S16. HMBC spectrum of narciabduliine (**2**); Figure S17. H2BC spectrum of narciabduliine (**2**); Figure S18. NOESY spectrum of narciabduliine (**2**); Figure S19. Stacked plot of dynamic NMR analysis of narciabduliine (**2**) at different temperatures (CDCl_3_).

## Sample Availability

Samples of the compounds are available from the authors.
