# Supplementary material for: Structure Elucidation and Cholinesterase Inhibition Activity of Two New Minor Amaryllidaceae Alkaloids
Source: Molecules. 2021 Feb 26;26(5):1279. doi: 10.3390/molecules26051279 (PMC7956344; doi:10.3390/molecules26051279)

Supplementary Material

Structure Elucidation and Cholinesterase Inhibition Activity of Two New Minor Amaryllidaceae Alkaloids

Jana Maříková[1], Abdullah Al Mamun[2], Latifah Al Shammari[2], Jan Korábečný[3,4], Tomáš Kučera[3], Daniela Hulcová[2,5], Jiří Kuneš[1], Milan Malaník[6], Michaela Vašková[7], Eliška Kohelová[2], Lucie Nováková[8], Lucie Cahlíková[2], and Milan Pour[1,*]

[1] Department of Bioorganic and Organic Chemistry, Faculty of Pharmacy, Charles University, Heyrovskeho 1203, 500 05 Hradec Kralove, Czech Republic; marikoj2@faf.cuni.cz (J.M.); kunes@faf.cuni.cz (J.K.)

[2] ADINACO Research Group, Department of Pharmaceutical Botany, Faculty of Pharmacy, Charles University, Heyrovskeho 1203, 500 05 Hradec Kralove, Czech Republic; almamuna@faf.cuni.cz (A.A.M.); alshamml@faf.cuni.cz (L.A.S.); kohelove@faf.cuni.cz (E.K.); cahlikova@faf.cuni.cz (L.C.)

[3] Department of Toxicology and Military Pharmacy, University of Defence, Trenesska 1575, 500 05 Hradec Kralove, Czech Republic; tomas.kucera2@unob.cz (T.K.)

[4] Biomedical Research Centre, University Hospital Hradec Králové, Sokolska 581, 500 05 Hradec Kralove, Czech Republic; jan.korabecny@fnhk.cz (J.Ko.)

[5] Department of Pharmacognosy, Faculty of Pharmacy, Charles University, Heyrovskeho 1203, 500 05 Hradec Kralove, Czech Republic; hulcovd@faf.cuni.cz (D.H.)

[6] Department of Natural Drugs, Faculty of Pharmacy, Masaryk University, Palackeho trida 1946/1, 612 00 Brno, Czech Republic; milan.malanik@seznam.cz (M.M.)

[7] Department of Chemistry, Faculty of Science, University of Hradec Kralove, Rokitanskeho 62, 500 03 Hradec Kralove, Czech Republic; michaela.vaskova@uhk.cz (M.V.)

[8] Department of Analytical Chemistry, Faculty of Pharmacy, Charles University, Heyrovskeho 1203, 500 05 Hradec Kralove, Czech Republic; novakoval@faf.cuni.cz (L.N.)

* Correspondence: pour@faf.cuni.cz; Tel.: +420 495 067 277

Table of contents

Figure S1. ESI-HRMS spectrum of 9-*O*-demethyllycorenine (**1**) 3

Figure S2. ECD data of 9-*O*-demethyllycorenine (**1**), and lycorenine 3

Figure S3. ^{1}H NMR spectrum of 9-*O*-demethyllycorenine (**1**) in $CDCl_3$ 4

Figure S4. ^{13}C NMR spectrum of 9-*O*-demethyllycorenine (**1**) in $CDCl_3$ 4

Figure S5. gCOSY spectrum of 9-*O*-demethyllycorenine (**1**) 5

Figure S6. gHSQC spectrum of 9-*O*-demethyllycorenine (**1**) 5

Figure S7. gHMBCAD spectrum of 9-*O*-demethyllycorenine (**1**) 6

Figure S8. gH2BC spectrum of 9-*O*-demethyllycorenine (**1**) 6

Figure S9. NOESY spectrum of 9-*O*-demethyllycorenine (**1**) 7

Figure S10. ESI-HRMS spectrum of narciabduliine (**2**) 7

Figure S11. ^{1}H NMR spectrum of narciabduliine (**2**) in $CDCl_3$ 8

Figure S12. ^{13}C NMR spectrum of narciabduliine (**2**) in $CDCl_3$ 8

Figure S13. gCOSY spectrum of narciabduliine (**2**) 9

Figure S14. gHSQC spectrum (aromatic region) of narciabduliine (**2**) 9

Figure S15. gHSQC spectrum (aliphatic region) of narciabduliine (**2**) 10

Figure S16. gHMBCAD spectrum of narciabduliine (**2**) 10

Figure S17. gH2BC spectrum of narciabduliine (**2**) 11

Figure S18. NOESY spectrum of narciabduliine (**2**) 11

Figure S19. Stacked plot of dynamic NMR analysis of narciabduliine (**2**) at different temperatures ($CDCl_3$) 12

Figure S1. ESI-HRMS spectrum of 9-*O*-demethyllycorenine (**1**)

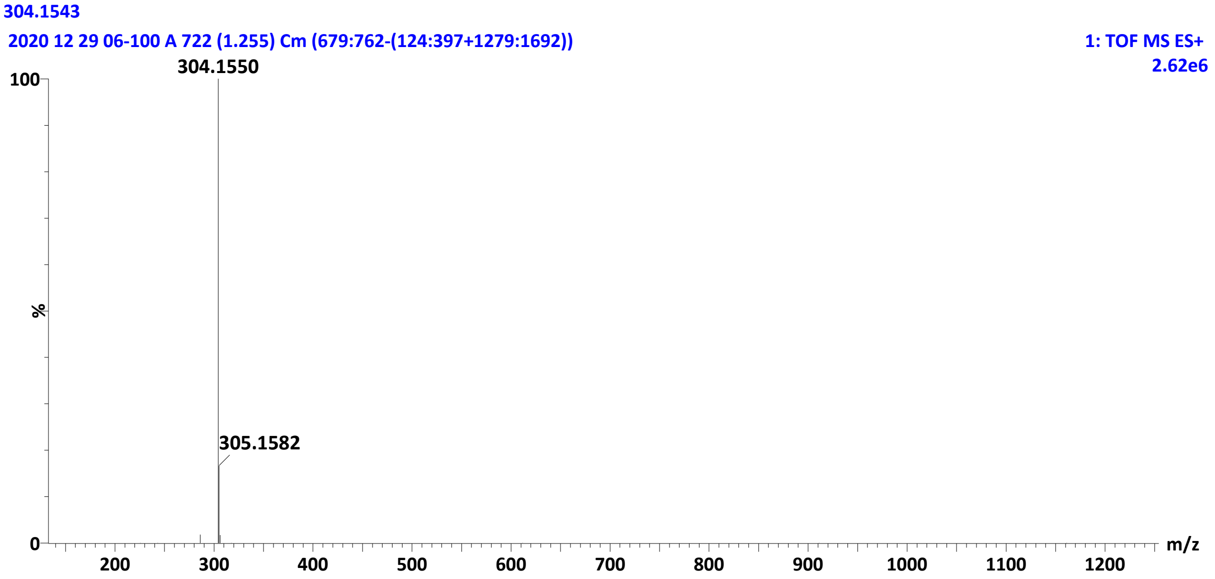

Figure S2. ECD data of 9-*O*-demethyllycorenine (**1**), and lycorenine

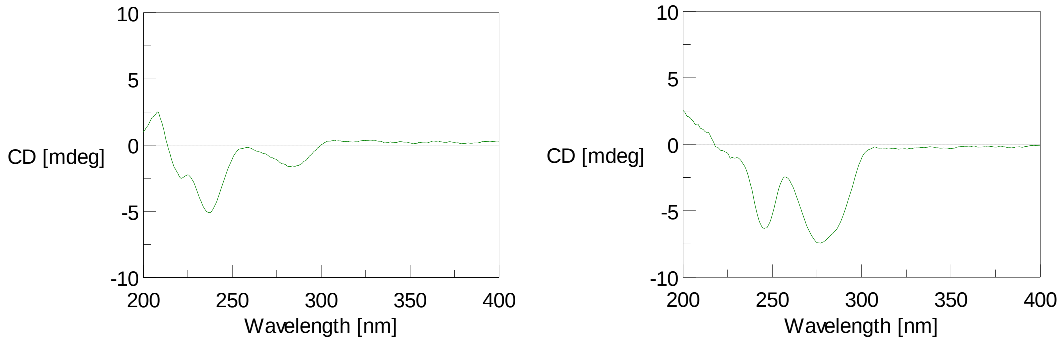

9-*O*-demethyllycorenine lycorenine

Figure S3. ^{1}H NMR spectrum of 9-*O*-demethyllycorenine (**1**) in $CDCl_3$

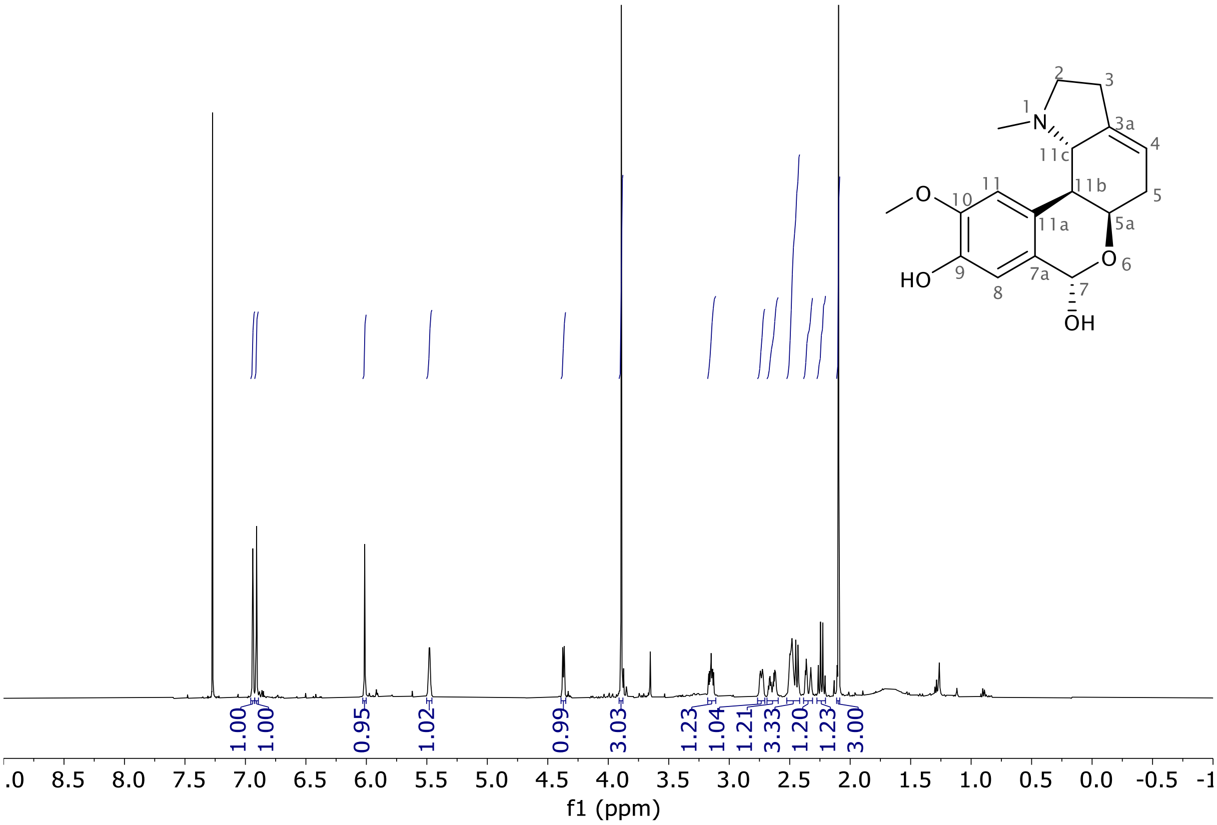

Figure S4. ^{13}C NMR spectrum of 9-*O*-demethyllycorenine (**1**) in $CDCl_3$

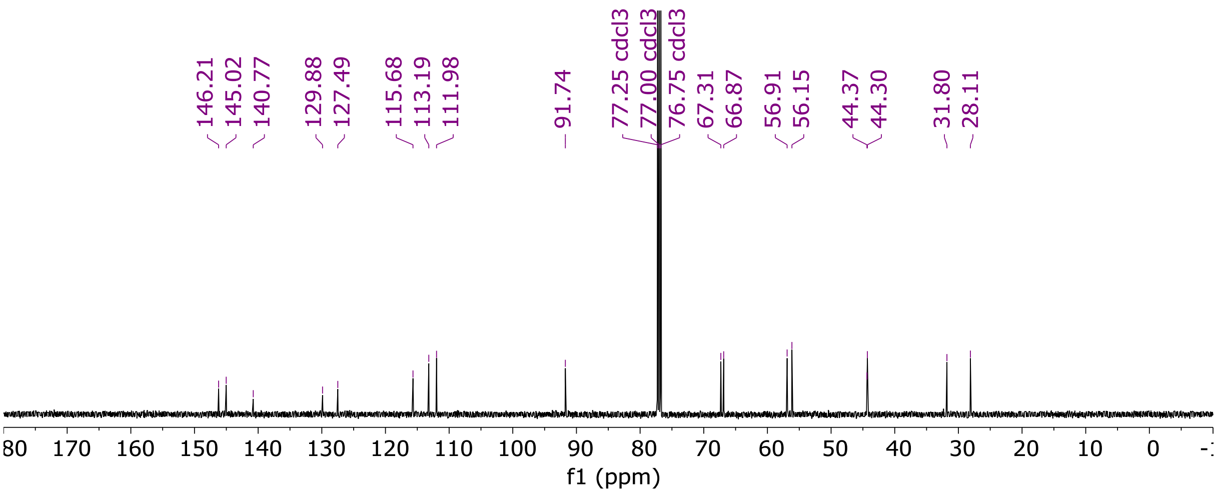

Figure S5. gCOSY spectrum of 9-*O*-demethyllycorenine (**1**)

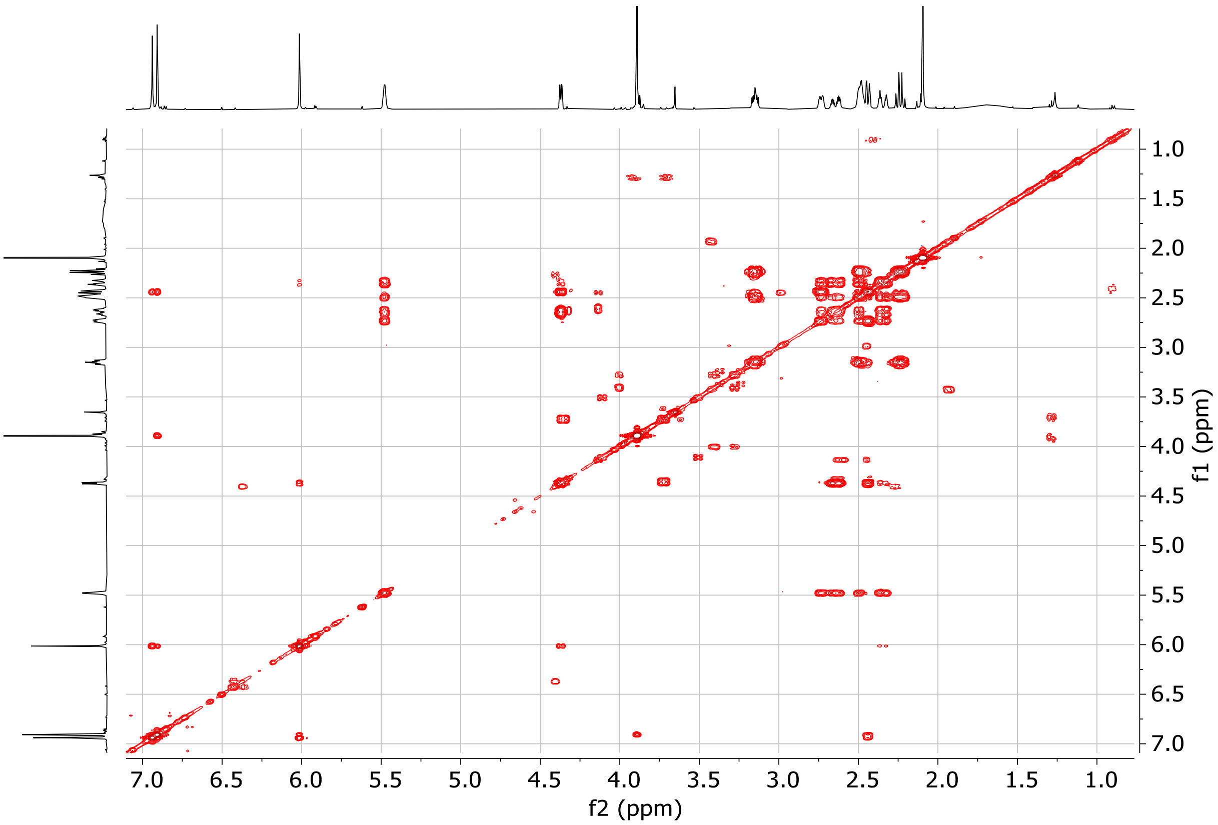

Figure S6. gHSQC spectrum of 9-*O*-demethyllycorenine (**1**)

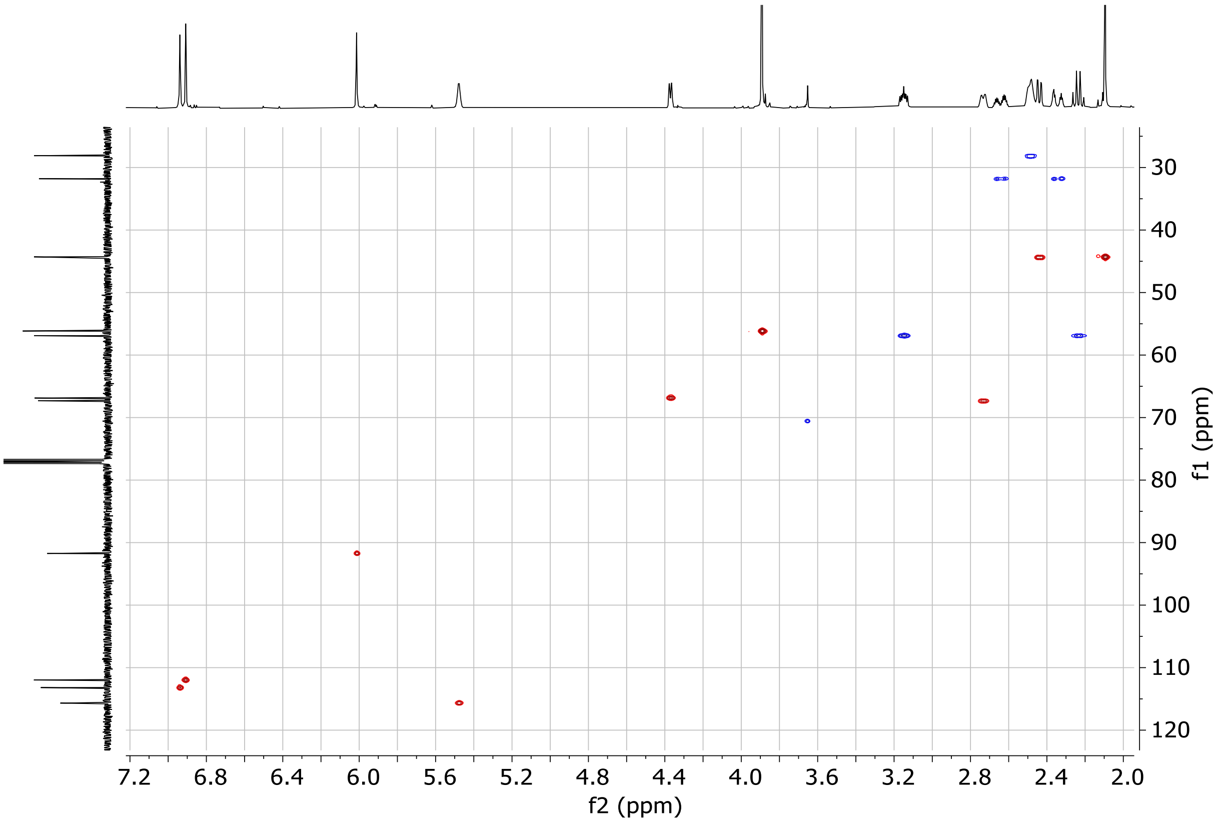

Figure S7. gHMBCAD spectrum of 9-*O*-demethyllycorenine (**1**)

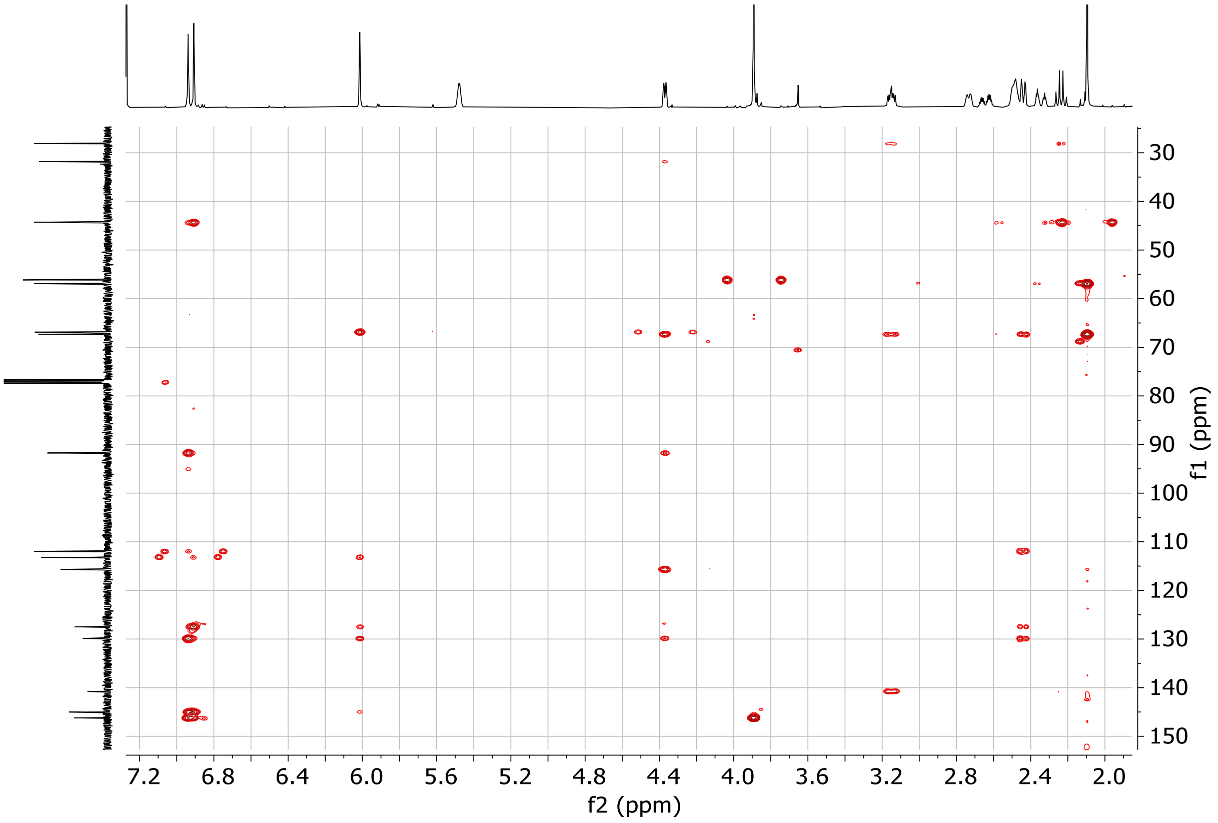

Figure S8. gH2BC spectrum of 9-*O*-demethyllycorenine (**1**)

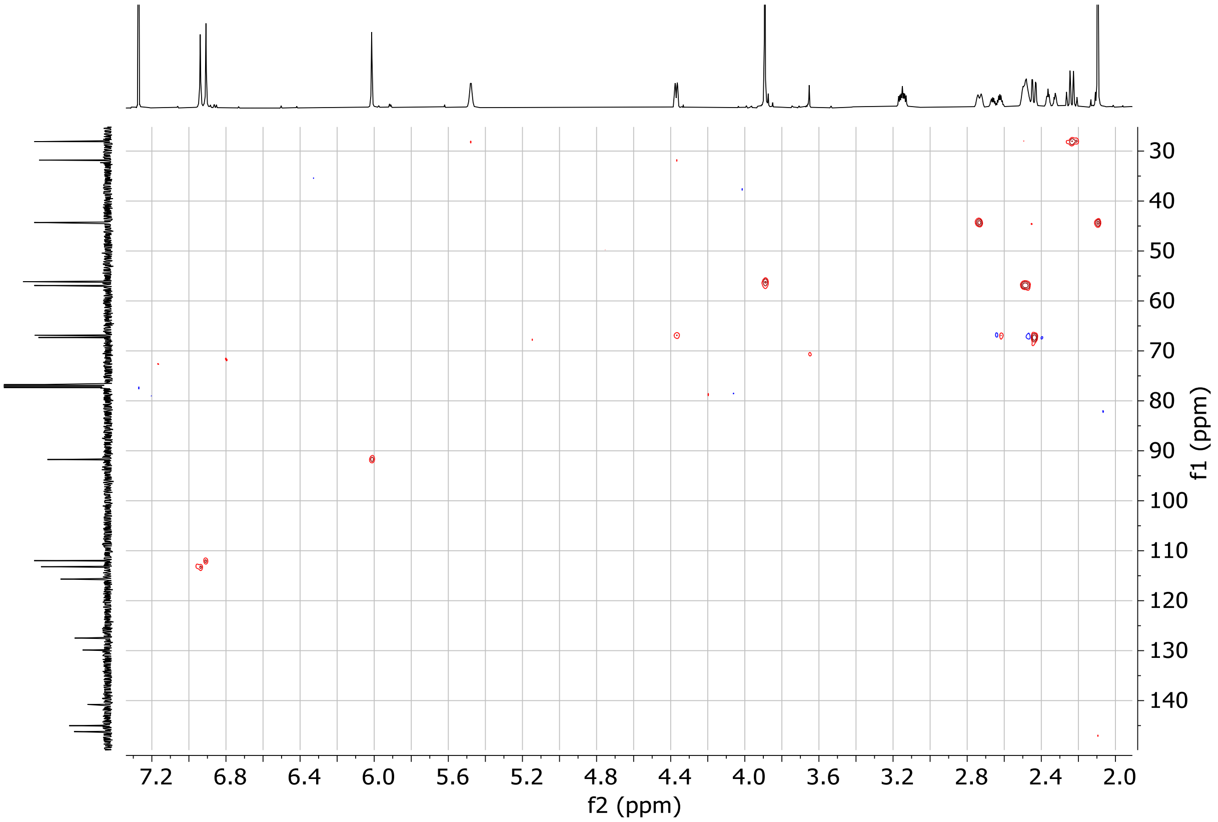

Figure S9. NOESY spectrum of 9-*O*-demethyllycorenine (**1**)

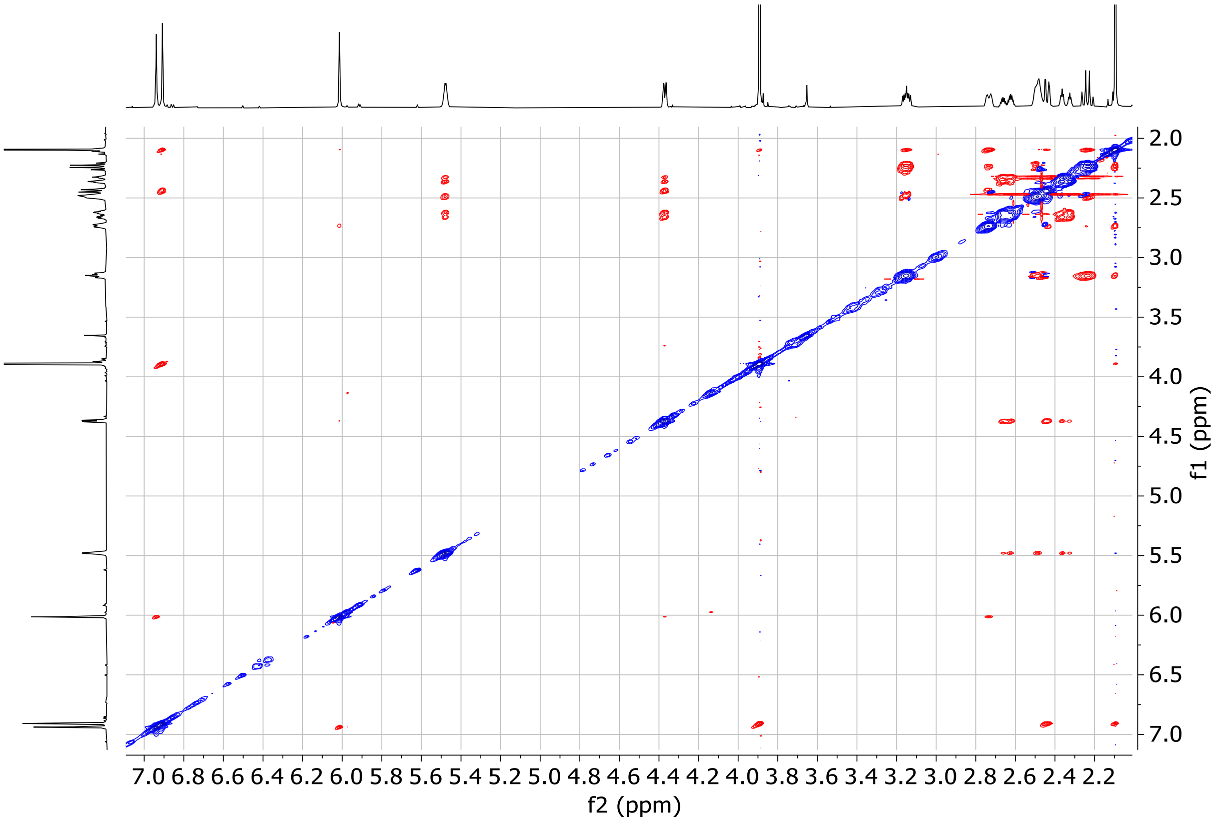

Figure S10. ESI-HRMS spectrum of narciabduliine (**2**)

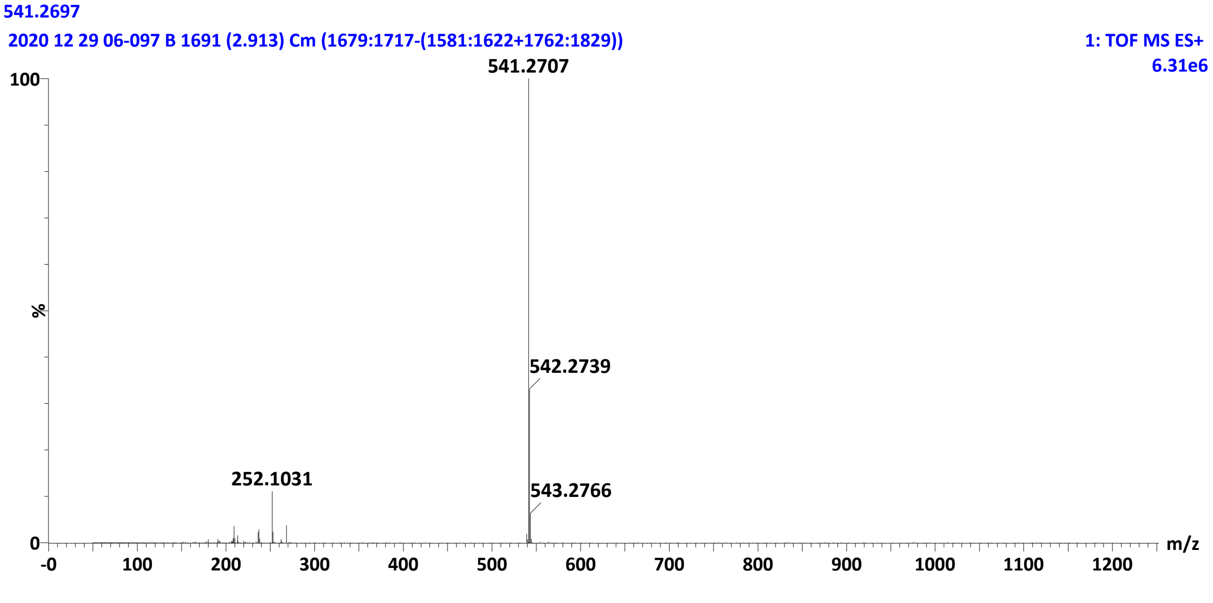

Figure S11. ^{1}H NMR spectrum of narciabduliine (**2**) in $CDCl_3$

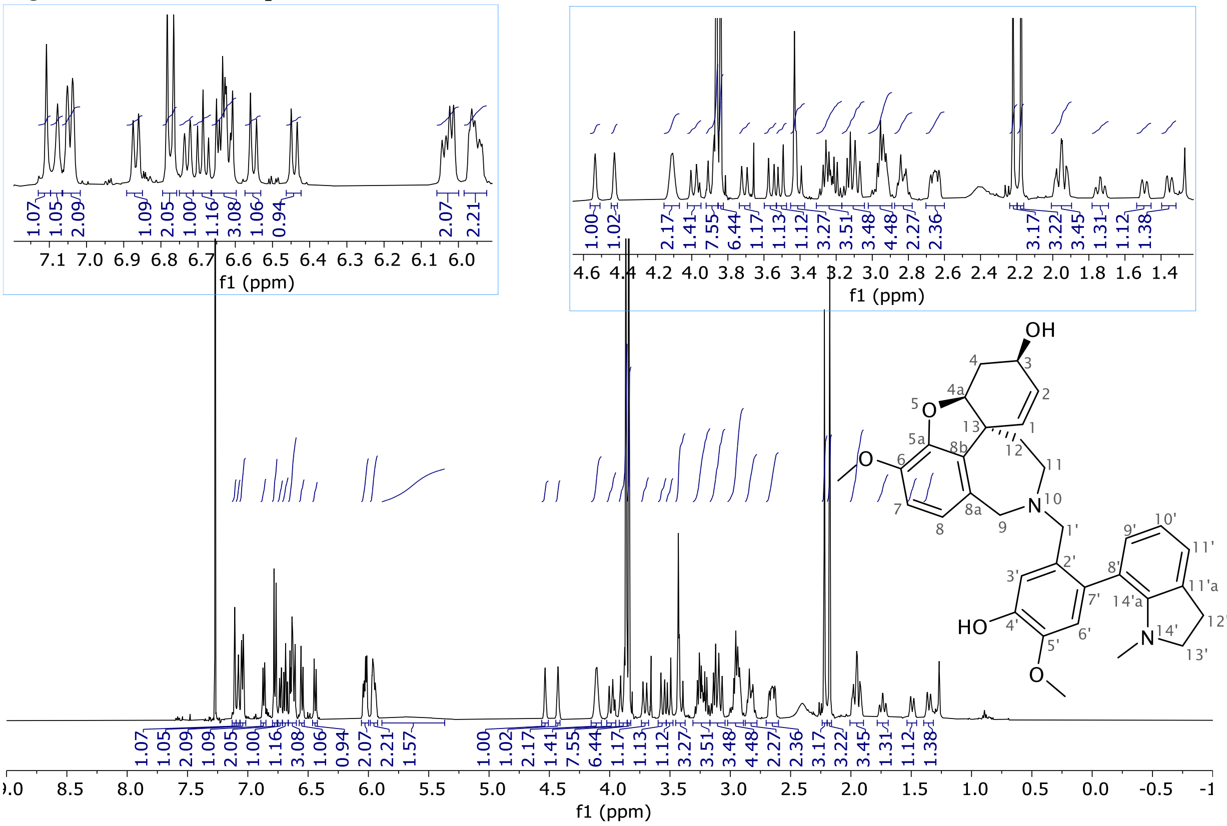

Figure S12. ^{13}C NMR spectrum of narciabduliine (**2**) in $CDCl_3$

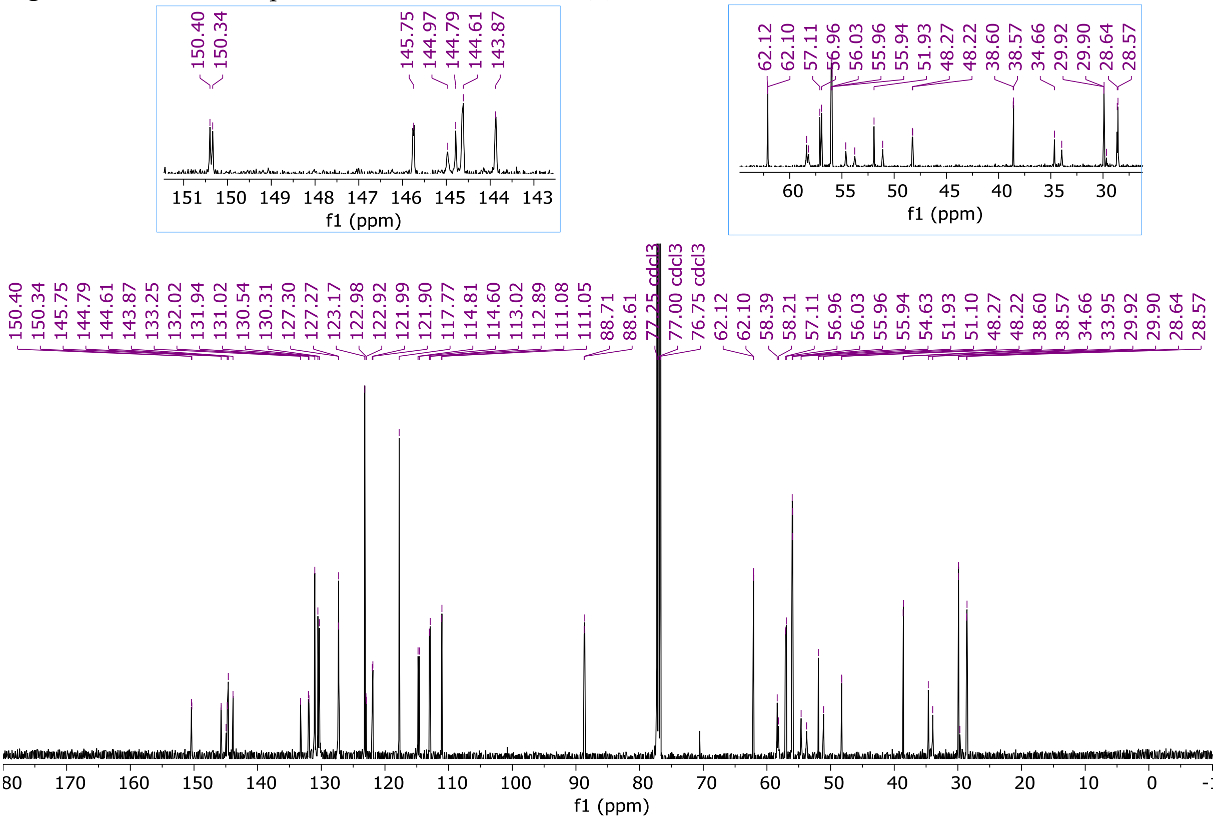

Figure S13. gCOSY spectrum of narciabduliine (**2**)

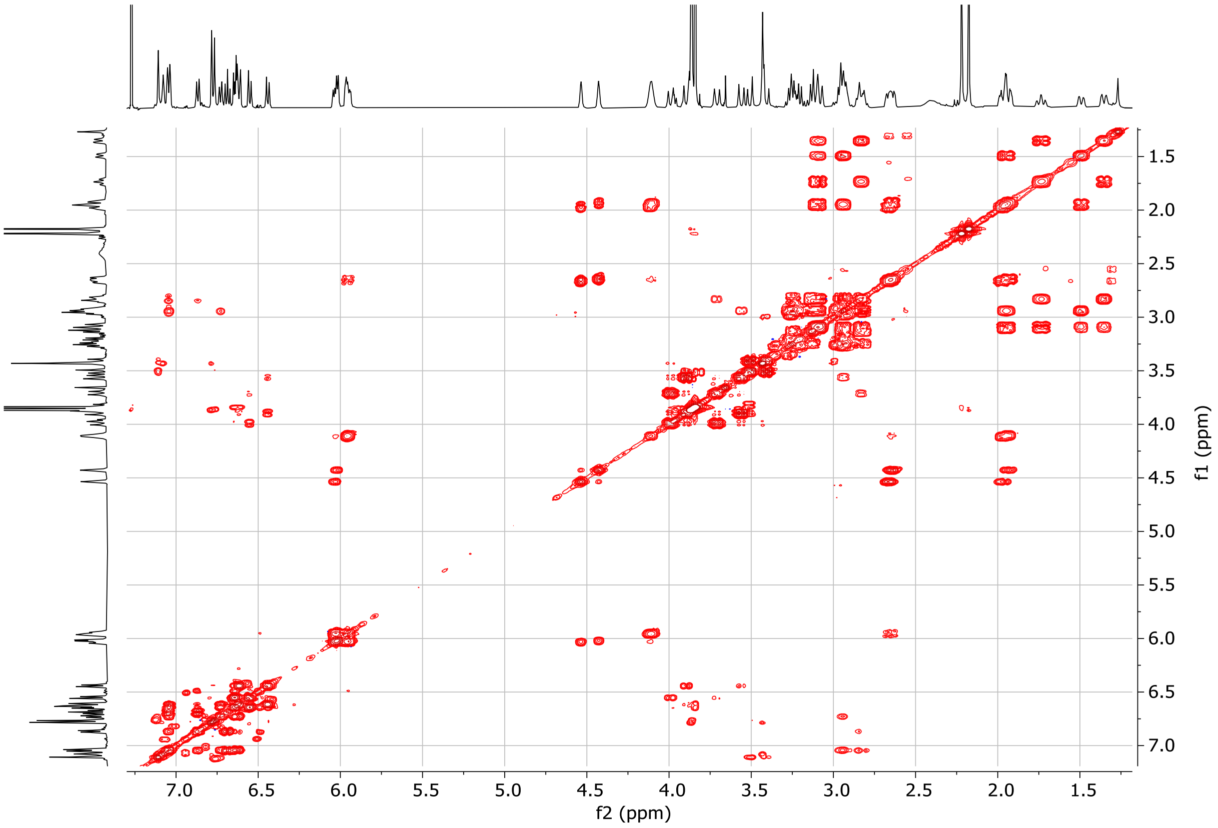

Figure S14. gHSQC spectrum (aromatic region) of narciabduliine (**2**)

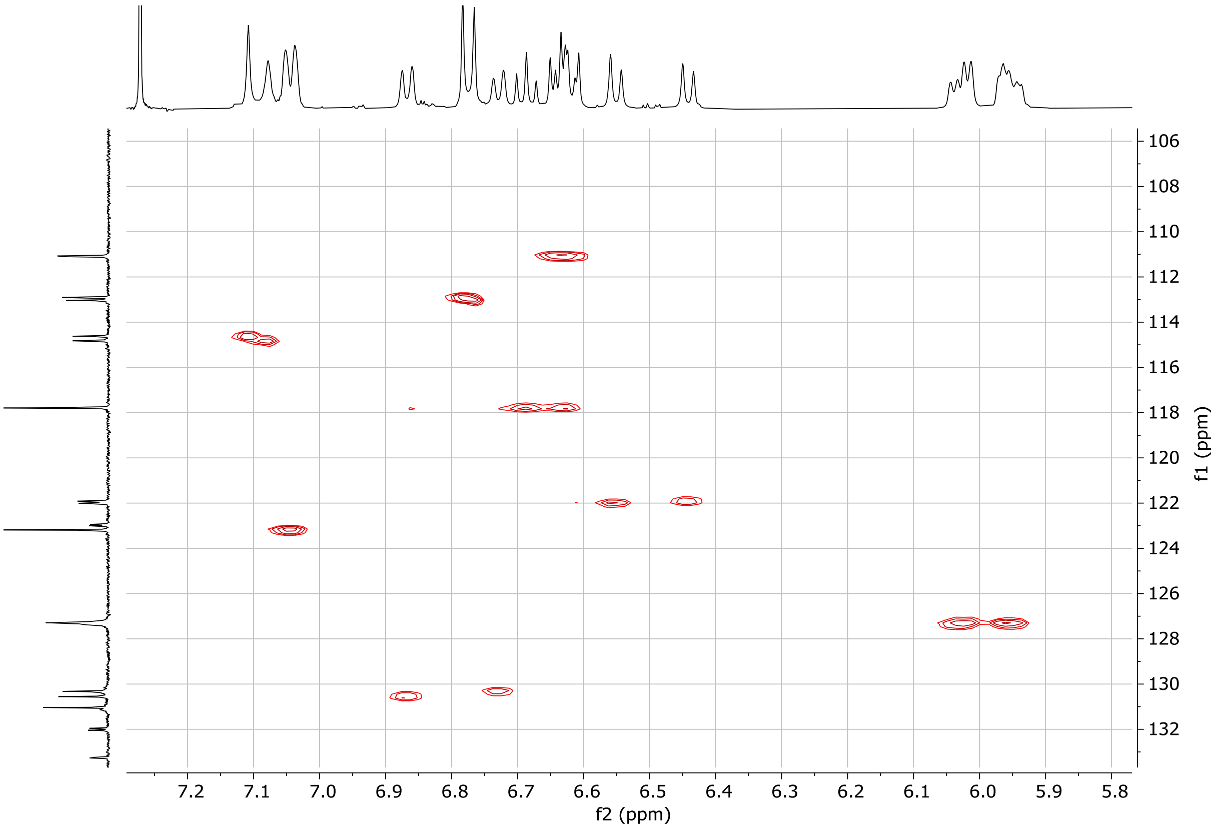

Figure S15. gHSQC spectrum (aliphatic region) of narciabduliine (**2**)

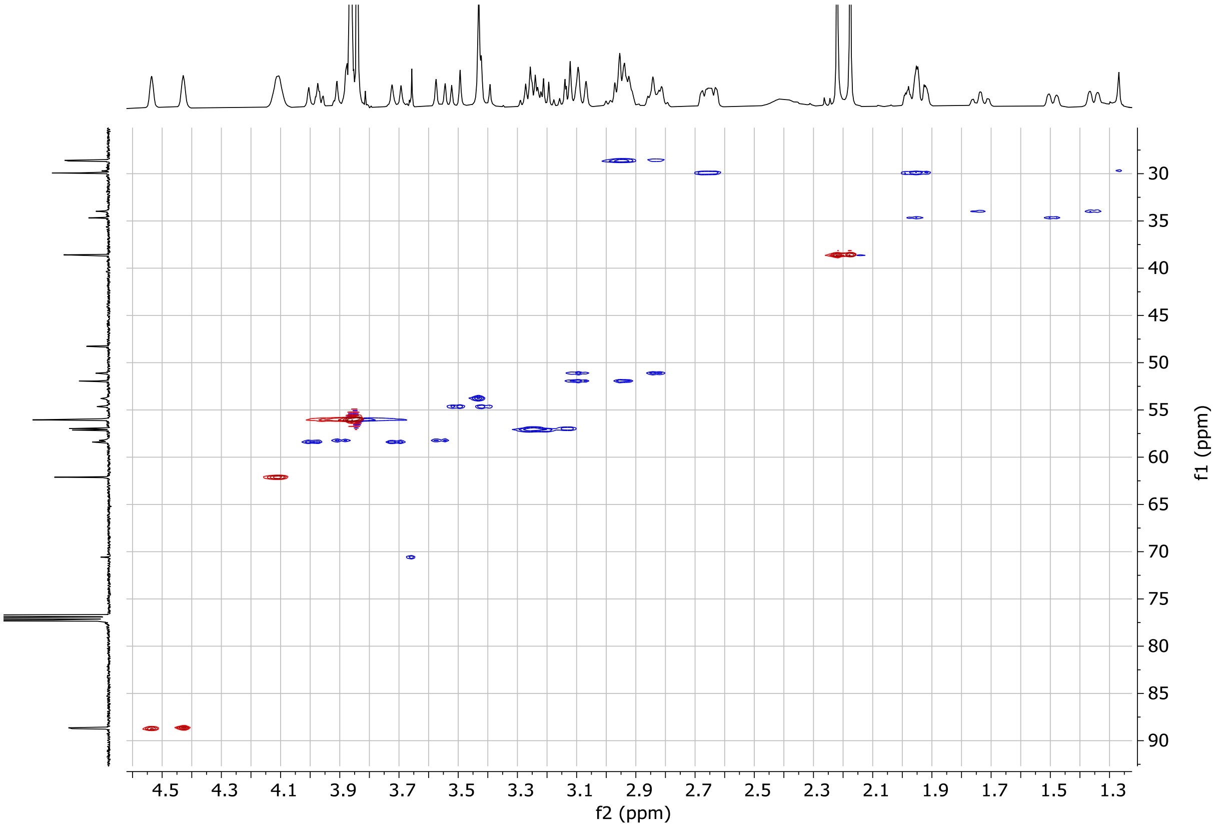

Figure S16. gHMBCAD spectrum of narciabduliine (**2**)

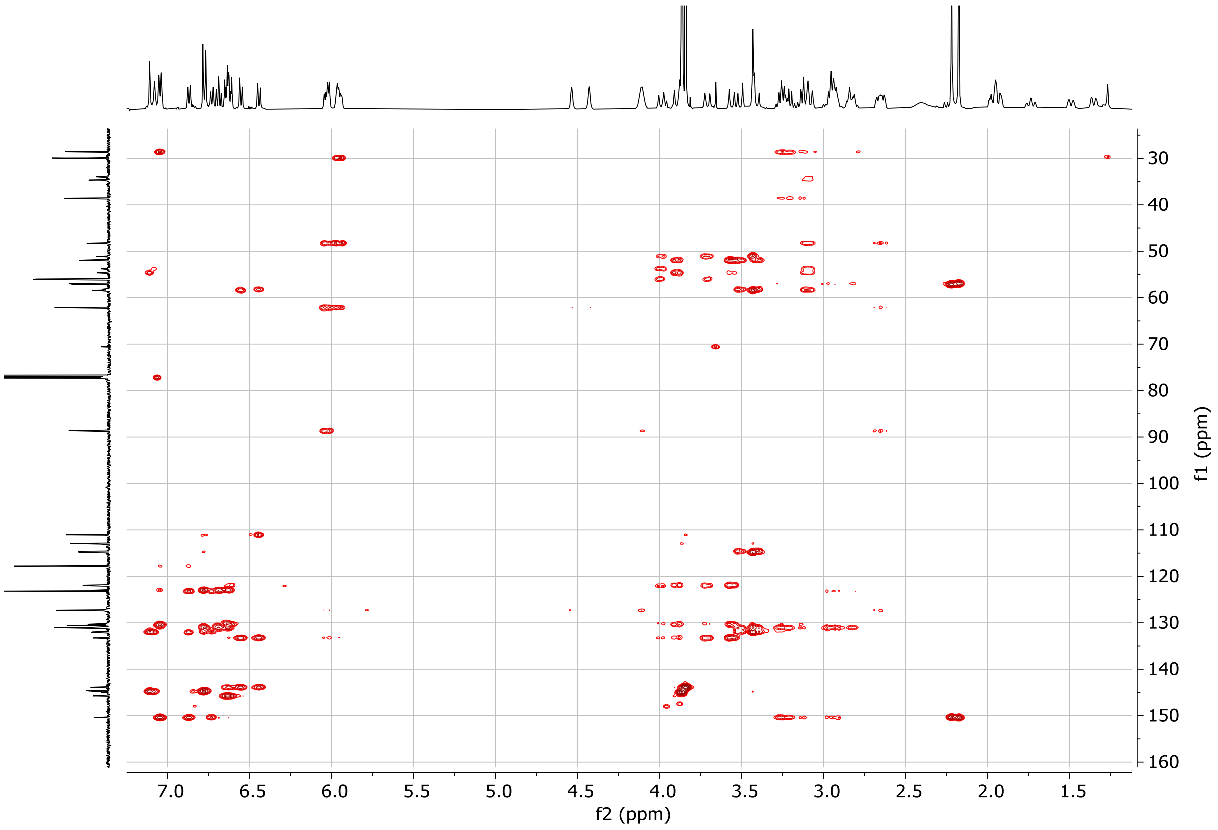

Figure S17. gH2BC spectrum of narciabduliine (**2**)

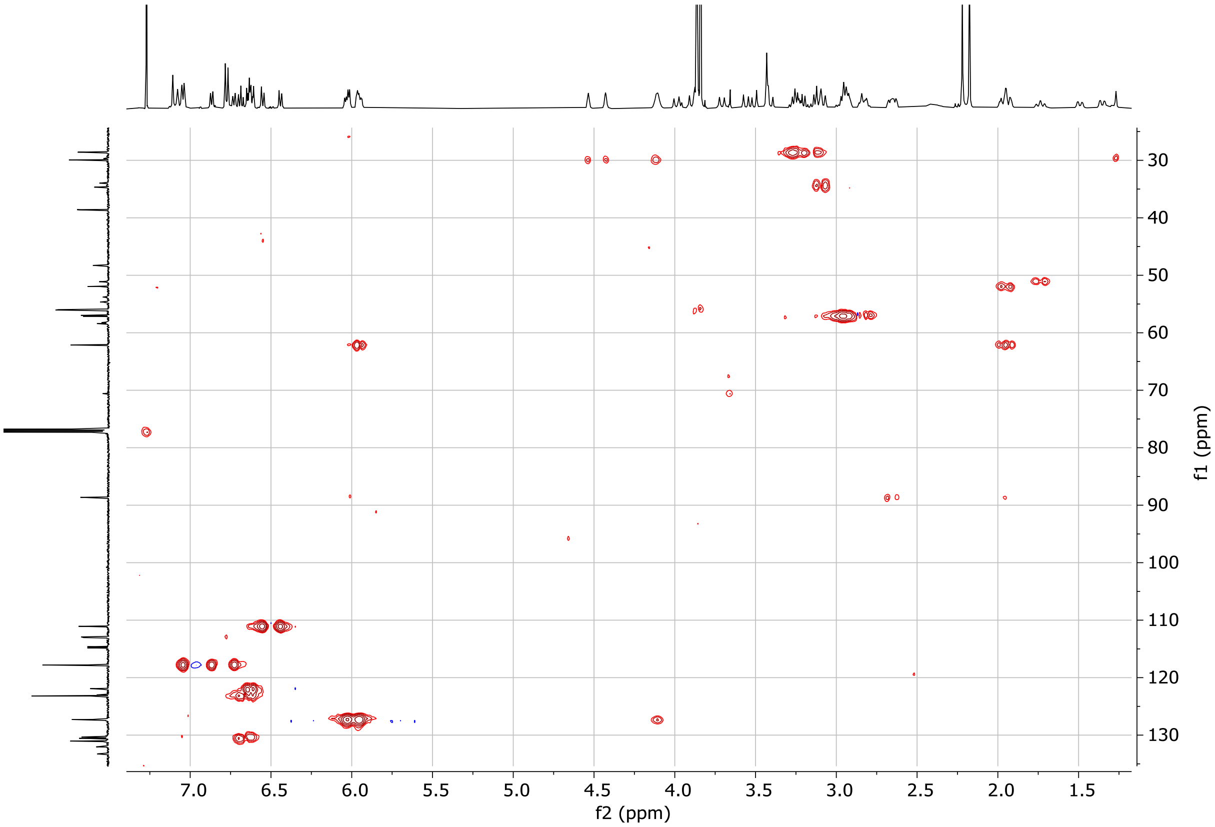

Figure S18. NOESY spectrum of narciabduliine (**2**)

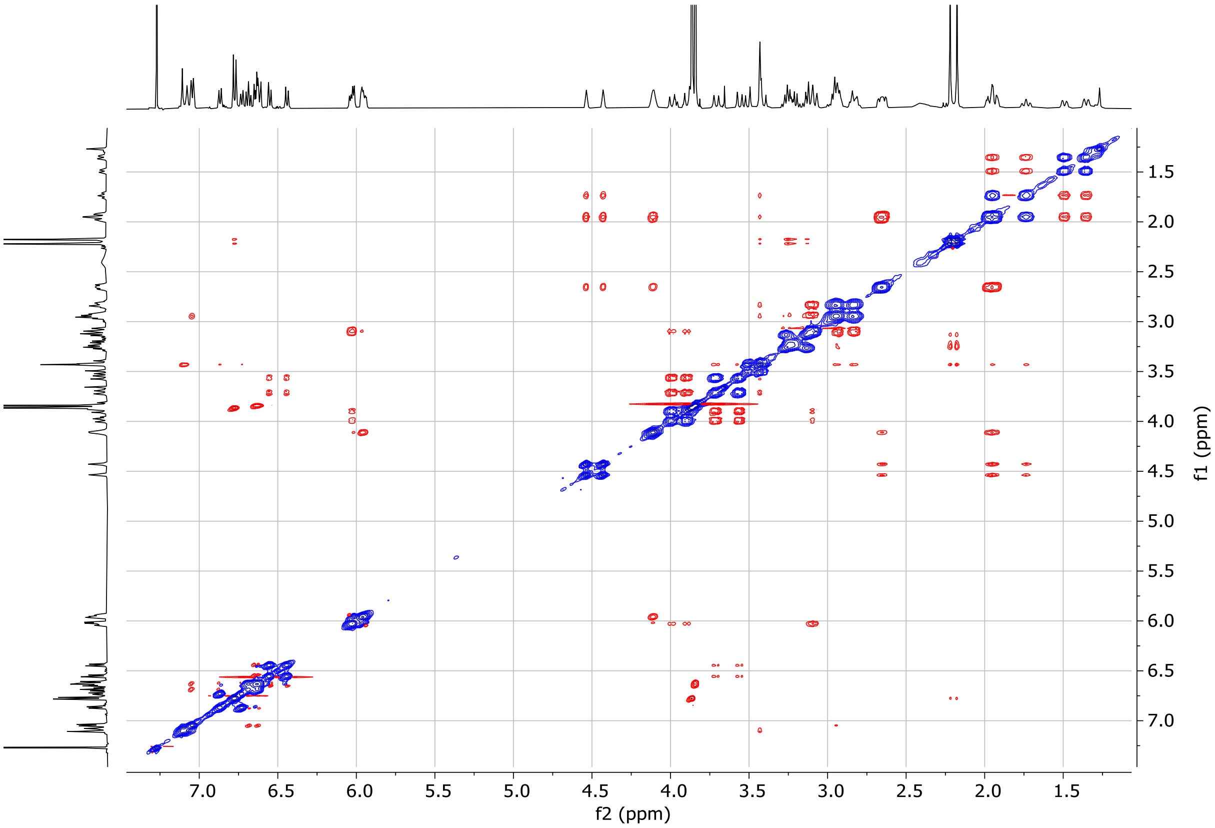

Figure S19. Stacked plot of dynamic NMR analysis of narciabduliine (**2**) at different temperatures ($CDCl_3$)

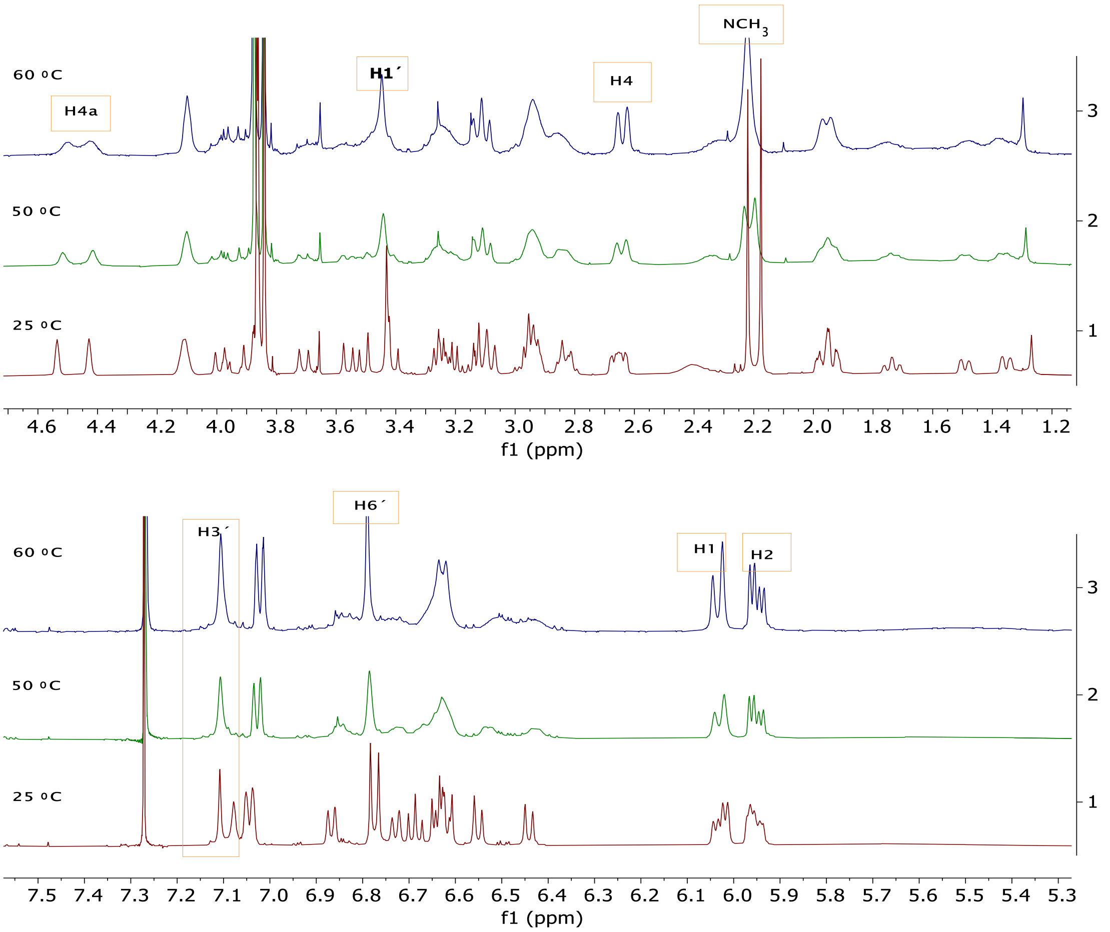

Supplement: Supplementary file 1 [file molecules-26-01279-s001.pdf]
